# Analysis of clinical pharmacist interventions in a tertiary teaching hospital in Brazil

**DOI:** 10.1590/S1679-45082013000200010

**Published:** 2013

**Authors:** Wálleri Christini Torelli Reis, Carolinne Thays Scopel, Cassyano Januário Correr, Vânia Mari Salvi Andrzejevski

**Affiliations:** 1 Hospital de Clínicas, Universidade Federal do Paraná, Curitiba, PR, Brazil; 2 Universidade Federal do Paraná, Curitiba, PR, Brazil

**Keywords:** Pharmacy service, hospital, Pharmaceutical care, Hospital pharmacy, Drug prescriptions

## Abstract

**Objective:**

To analyze the clinical pharmacist interventions performed during the review of prescription orders of the Adult Intensive Care, Cardiologic Intensive Care, and Clinical Cardiology Units of a large tertiary teaching hospital in Brazil.

**Methods:**

The analysis took place daily with the following parameters: dose, rate of administration, presentation and/or dosage form, presence of inappropriate/unnecessary drugs, necessity of additional medication, more proper alternative therapies, presence of relevant drug interactions, inconsistencies in prescription orders, physical-chemical incompatibilities/solution stability. From this evaluation, the drug therapy problems were classified, as well as the resulting clinical interventions.

**Results:**

During the study, a total of 6,438 drug orders were assessed and 933 interventions were performed. The most prevalent drug therapy problems involved ranitidine (28.44%), enoxaparin (13.76%), and meropenem (8.26%). The acceptability of the interventions was 76.32%. The most common problem found was related to dose, representing 46.73% of the total.

**Conclusion:**

Our study showed that up to 14.6% of the prescriptions reviewed had some drug therapy problem and the pharmacist interventions have promoted positive changes in seven to ten of these prescriptions.

## RESUMO

**Objetivo:**

Analisar as intervenções realizadas por farmacêuticos clínicos durante a revisão de prescrições médicas das Unidades de Terapia Intensiva Adulto, Terapia Intensiva Cardiológica e de Cardiologia Clínica de um hospital universitário terciário do Brasil.

**Métodos:**

A análise de prescrições foi realizada diariamente com avaliação dos seguintes parâmetros: dose, intervalo de administração, apresentação e/ou forma farmacêutica, presença de medicamentos inapropriados/desnecessários, necessidade de medicamento adicional, alternativas terapêuticas mais adequadas, presença de interações medicamentosas relevantes, inconsistências nas prescrições, incompatibilidades físico-químicas/estabilidade da solução. A partir dessa avaliação, os problemas relacionados aos medicamentos foram classificados, bem como as intervenções farmacêuticas resultantes, conforme estabelecido pelo manual de farmácia clínica do hospital.

**Resultados:**

Durante o estudo, um total de 6.438 prescrições foi avaliado e foram realizadas 933 intervenções farmacêuticas. Os medicamentos mais envolvidos nos problemas foram: ranitidina (28,44%), enoxaparina (13,76%) e meropenem (8,26%). A aceitação das intervenções foi de 76,32%. O problema mais comumente encontrado foi relacionado à dose, representando 46,73% do total.

**Conclusão:**

Até 14,6% das prescrições avaliadas apresentaram algum problema relacionado a medicamentos. As intervenções farmacêuticas promoveram mudanças positivas em sete a cada dez dessas prescrições.

## INTRODUCTION

The irrational use of medication is a major worldwide public health problem, with a great impact on clinical, economic, and humanistic outcomes. It is estimated that prescription errors can lead to an increase of 50 to 70% in the government funds for medication. However, when used properly, medications are the most cost-effective therapeutic resources^(1,2)^.

The rational use of medication occurs when patients receive the appropriate medication for their clinical need, at the correct dosage, for a suitable period of time, and at the lowest cost to them and to the community. In this context, the following processes are included: appropriate pharmacotherapy, appropriate indication, appropriate medication, right dose according to the patient's clinical condition, appropriate administration and duration of treatment, appropriate patient, patient's adherence to treatment, and monitoring of the outcome of pharmacotherapy, as well as monitoring and evaluation of possible adverse drug-to-drug reactions related to the treatment^(2)^.

The publication of the report *To err is human: building a safer health system* by the Institute of Medicine, in 1999, showed that the health care provided to patients is not as safe as it should be and that many deaths occur every year due to medication errors, including prescription errors, thus emphasizing the importance of measures to ensure the safety and rational use of medication, pointing to the need of involvement and mobilization of the multi-professional staff^(3)^. It has been estimated, by the World Health Organization (WHO), that more than 50% of all medications are prescribed, dispensed, or sold inappropriately^(4)^.

Studies have shown that prescription orders are involved in most of the cases of medication error. In the analysis of 4,031 patient records at two teaching hospitals in the United States, 49% of them were associated to prescription errors^(5,6)^. Likewise, systematic reviews have shown that on average 7 to 10% of prescriptions have some type of error^(7,8)^.

The activities developed by the clinical pharmacist play a key role in promoting better medication use, ensuring that patients receive appropriate pharmacotherapy, thus minimizing the risk of unfavorable outcomes of pharmacotherapy and consequently reducing costs^(2,9,10)^. Among these activities, the review of medication orders is extremely important, and it enables identifying, solving and preventing the emergence of drug therapy problems (DTP) and negative outcomes associated with medication^(11)^.

## OBJECTIVE

The aim of this study was to analyze the clinical pharmacist interventions performed during the review of prescription orders of the Adult Intensive Care, Cardiologic Intensive Care, and Clinical Cardiology Units of a large tertiary teaching hospital in Brazil.

## METHODS

This was a prospective study of clinical pharmacist interventions (CPI) and identification of DTP performed during the review of prescription orders in the Hospital Pharmacy Unit of *Hospital de Clínicas da Universidade Federal do Paraná* (HC-UFPR). The project was approved by the Ethics Committee of the hospital in February 28, 2012 with number CAAE 00883912.0.0000.0096.

The systematization of the clinical pharmacy service began with a literature review and development of a work proposal. Subsequently, meetings were held with the participation of residents, the manager of the Hospital Pharmacy Unit, residence mentoring and preceptorship to define the priority action plan and to establish a work methodology to guide the activities of clinical pharmacists.

The selection of the inpatient care units for the implementation of clinical activities by pharmacists was based on the analysis of the demands recorded in the medication dispensing section, and on the data collected from clinical interventions performed by pharmacists in 2010. Another aspect to guide this choice was the area of concentration offered by the residency programs in hospital pharmacy.

From these data, a strategy to approach the heads of clinical units was designed in order to present the clinical pharmacy service and permit the beginning of a relationship based on trust and knowledge sharing between the teams. The presentation of the service occurred through face-to-face meetings and group discussions.

Clinical activities started with a daily analysis of the prescriptions by the pharmacists. In HC-UFPR, prescription orders are validated every 24 hours, with defined schedules for each inpatient unit, and it is not possible to dispense drugs without electronic prescription. After that, clinical pharmacists evaluated the orders and the drugs were subsequently dispensed by pharmacy technicians. It is important to note that each clinical pharmacist accompanied a defined inpatient care unit, evaluating medical prescriptions, participating in multiprofessional clinical rounds, and interacting with the healthcare team and with patients, whenever possible. Thus, in our context, the clinical pharmacist was responsible for monitoring the pharmacotherapeutic needs of patients, seeking to guarantee the rational and safe use of drugs.

Data collection for the study was conducted from July 2011 to July 2012, in the Adult Intensive Care Unit (ICU), Cardiologic Intensive Care Unit and Clinical Cardiology Unit.

Prescription order review consisted of an assessment by the pharmacist of parameters related to medication selection, therapeutic regimen, and administration instructions. Regarding the choice of the classification method of DTP and CPI, several references were consulted, despite the fact that most of them presented limitations in their application to the reality of the hospital. Therefore, we opted to design a methodology applied to our local reality, based on *Tercer Consenso de Granada, Manual para la Atención Farmacéutica* proposed by Clemente Martí and Jiménez-Torres^(12)^ and on the recommendations of the American College of Clinical Pharmacy and American Society of Health-System Pharmacists^(11–14)^.

During the prescription review process, the pharmacist had access to the following databases: Drugdex^®^, UpToDate^®^ and Medscape^®^. Each parameter of evaluation was considered as follows:

– dose: evaluating if the dose prescribed is recommended in accordance with the literature, considering weight or body surface of the patient, and the necessity for adjustment for altered renal and/or hepatic function;
– medication interval: evaluating if the intervals of administration of prescribed medications were described in the literature, and the possible suitability of the same for abnormal kidney and liver function, also considering the possibility of reducing costs and time spent by nursing administration;
– route of administration: evaluating the route of administration, based on pharmacokinetic characteristics and patient clinical condition;
– presentation and/or dosage form: evaluating if the hospital standardization is adequate according to the patient (children, elderly, patients with swallowing problems or feeding tube);
– inappropriate/unnecessary medication: evaluating if there is any medication without indication for the clinical condition, therapeutic duplicity, counter-indicated or unnecessary medication for the clinical condition of the patient, and patient with known allergic reaction to medication;
– necessity of additional medication: evaluating if there is any untreated medical condition, continued treatment, prophylactic or preventive medication;
– more appropriate and/or alternative therapy available: evaluating if there is a medication, more effective, cost-effective or safer and available in the hospital standard medication list;
– drug interactions: evaluating if there is any drug-to-drug interaction with clinical relevance according to the classifications found in the databases;
– inconsistencies in prescription orders: discrepant information about dosing or administration instructions contained in the same medication order;
– dilution and/or infusion rate: evaluating the concentration and infusion rate of the medication;
– physical-chemical incompatibilities and/or preparation stability: evaluating possible incompatibilities between drugs and drug/diluent and verification of the stability of the medications prescribed in accordance with the standard dilution of each clinic.

When a DTP was identified during the prescription review, the system adopted by the pharmacist was to contact the physician or other health care professional responsible for the patient to discuss the best approach to take.

The DTP, the CPI and the acceptability were recorded and classified in standardized forms, and then tabulated in spreadsheets and sequentially analyzed. The acceptability of interventions was classified as follows: accepted; not accepted with justification, when the intervention was not accepted but there was a plausible explanation to justify the medical decision; not accepted without justification; accepted with alterations, in these cases an intervention was proposed, however during the discussion with the healthcare professional some change was made; does not apply to interventions consisting of educational actions.

As a way to establish a cycle for the improvement of existing processes, reports presenting the data obtained from the clinical pharmaceutical activities were periodically sent to the physicians responsible for the hospital units. Then, meetings were scheduled for to assess, discuss, and define continuous improvement actions.

## RESULTS

During the study period, 6,438 medication orders of over 1,000 patients were reviewed. The three units where patients came from (Clinical Cardiology Unit, Adult ICU, and Cardiologic ICU) have 15, 14, and 8 beds, respectively. All units have integrated multiprofessional teams in their activities, comprising the following professionals: physicians, nurses, pharmacists, nutritionists, psychologists, physiotherapists, and occupational therapists.

Among the study population, 53.29% of patients were male. The median age was 59 years and the average length of stay in clinical units was 4.61 days. About nine out of ten patients had some type of co-morbidity, the most common ones were hypertension (36.44%), coronary artery disease (23.27%), and *diabetes mellitus* (15.40%).

Eleven drugs on average were reviewed per prescription, and the average time required for evaluation of each prescription was 14.2 minutes. We found 933 DTPs, involving 129 drugs, in 247 working days, representing 3.78 problems per day of work.

The types of DTP found and frequencies are shown in the table 1. The problems found were: dose for 46.73% (n=436), inappropriate/unnecessary medication for 19.08% (n=178), more appropriate and/or available alternative therapy for 7.82% (n=73), interactions for 7.50% (n=70), presentation and/or pharmaceutical form for 6.86% (n=64), need of additional medication for 5.25% (n=49), inconsistencies in prescription orders for 3.32% (n=31), and medication interval for 2.89% (n=27). Physical-chemical incompatibilities and/or preparation stability (n=3), route of administration (n=1), and dilution and/or infusion rate (n=1) showed percentages lower than of 1%.

**Table 1:** Drug therapy problem

DTP	n (%)
Dose	436 (46.73)
Dosing interval	27 (2.89)
Route of administration	1 (0.11)
Presentation and/or pharmaceutical form	64 (6.86)
Inappropriate/unnecessary drug	178 (19.08)
Necessity of additional medication	49 (5.25)
More appropriate and/or available alternative therapy	73 (7.82)
Drug interactions	70 (7.50)
Prescription orders inconsistencies	31 (3.32)
Dilution and/or infusion rate	1 (0.11)
Physical-chemical incompatibilities and/or preparation stability	3 (0.32)
Total	933

DTP: drug therapy problem.

The therapeutic categories involved in DTPs are described in table 2. A closer look at the DTP dose demonstrates that the most prevalent medications were: ranitidine (n=124; 28.44%), followed by enoxaparin (n=60; 13.76%), and meropenem (n=36; 8.26%). In case of presence of DTP related to inappropriate/ unnecessary medication, there was a more uniform distribution of 77 medications involved and the most prevalent were enoxaparin (n=9; 5,06%), propofol (n=7; 3.93%), and ketoprofen (n=7; 3.93%). Considering the overview of problems encountered, ranitidine (n=140; 15.01%) and enoxaparin (n=83; 8.90%) were also the most prevalent medications.

**Table 2:** Drugs involved in drug therapy problems classified through the groups of the anatomical therapeutic chemical classification system

ATC category	n (%)*
Alimentary tract and metabolism	252 (27.01)
Blood and blood forming organs	161 (17.26)
Cardiovascular system	95 (10.18)
Dermatologicals	12 (1.29)
Genito-urinary system and sex hormones	1 (0.11)
Systemic hormonal preparations, excluding sex hormones and insulins	42 (4.50)
Anti-inflammatory for systemic use	192 (20.58)
Antineoplastic and immunomodulating agents	6 (0.64)
Musculo-skeletal system	7 (0.75)
Nervous system	128 (13.72)
Antiparasitic products, insecticides and repellents	4 (0.43)
Respiratory system	10 (1.07)
Sensory organs	0 (0)
Various	23 (2.47)
Total	933 (100)

ATC: anatomical therapeutic chemical.

As shown in the table 3, the CPIs performed were classified as: 50.38% (n=470) individualize/correct the dosing; 18.97% (n=177) suspend medication; 8.04% (n=75) replace by safer, more effective, more cost-effective and/or available presentation/pharmaceutical form; 7.50% (n=70) replace by safer, more effective, cost-effective and/or available medication; 6,43% (n=60) provide information/education to healthcare professionals; 4.93% (n=46) start medication; 3.22% (n=30) correct order error (recommendation/prescription); and 0,54% (n=5) correct preparation and/or administration by nursing team.

**Table 3:** Pharmaceutical clinical interventions

Pharmaceutical clinical interventions	n (%)	Examples
Suspend drug	177 (18.97)	Enoxaparin treatment dose in patient with active bleeding; two prescriptions of midazolam in the same order with different dose; omeprazole and ranitidine prescribed in the same order
Replace by safer, more effective and/or cost-effective drug	70 (7.50)	Replace omeprazole by ranitidine for stress ulcer. The cost of omeprazole is greater than ranitidine and the efficacy of the prophylaxis is equivalent
Replace by safer, more effective, more cost-effective and/or available presentation/ pharmaceutical form	75 (8.04)	Replace sublingual isosorbide dinitrate 5mg 3 times daily by immediate release isosorbide dinitrate 10mg 3 times daily; the sublingual presentation has a lower time of action than another to treat angina
Start drug	46 (4.93)	Suggest starting polystyrene sulfonate in a patient with hyperkalemia.
Individualize/correct the posology	470 (50.38)	Patient with clearance of creatinine (CrCl) <30mL/min, using enoxaparin. It is recommend to use 50% of the usual dose reported in literature.
Correct preparation and/or administration by nursing	5 (0.54)	It is recommend that ceftriaxone and calcium gluconate are not administered together in Y catheter, due the high risk of chemical interaction
Correct order errors (recommendation/prescription)	30 (3.22)	Prescription present insulin NPH 30UI in the morning but the order recommendation present insulin NPH 40UI
Provide information/education to healthcare professionals	60 (6.43)	Patient was taking clarithromycin and amitriptyline, this association may prolong the QT interval. Give information to the responsible physician to improve monitoring toxicity signals
Total	933	

**Figure 1:**
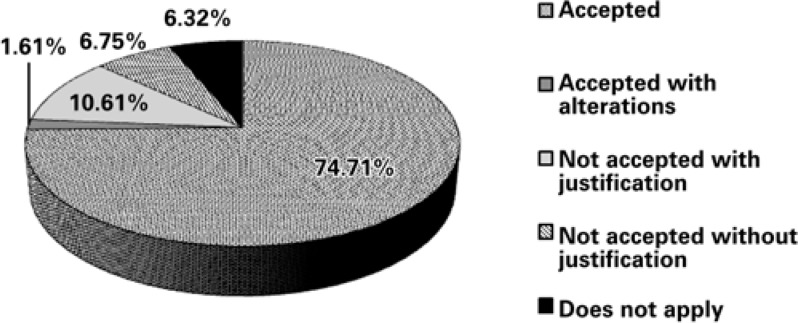
Acceptability of the pharmaceutical clinical interventions

Regarding the acceptability of CPIs, 74.71% (n=697) of the interventions were accepted, 10.61% (n=99) were not accepted with justification, 6.75% (n=63) were not accepted without justification, 1.61% (n=15) were accepted with alterations, and 6.32% (n=59) of the cases were included in the code “does not apply” (Figure 1).

## DISCUSSION

The multi-professional teamwork is the best way to ensure patient safety. During many years, Brazilian pharmacists were restricted to the management of hospital pharmacies; however, every day, the need of this professional in clinical units is becoming clearer and clearer. Several authors have shown that pharmacotherapy monitoring can reduce the rates of medication errors by up to 78%^(15–17)^. Our study showed that the review of prescription orders, integrated to the hospital dispensing routine, is an important way to detect and resolve medication errors and to improve the quality of medication use.

In hospitals, prescription orders play a key role in promoting the communication between the healthcare team and accounts for ensuring the correct use of medication. Considering this and that the review process of prescription orders is essential to improve pharmacotherapy to patients, particularly in hospitals, this activity was defined as priority. Moreover, studies showed that most of the medication errors occur during medication prescription and administration processes; so pharmacists could have a greater influence in the proper prescription towards quality in medication use^(5,6)^.

Prescription errors are a major cause of preventable adverse drug events, therefore interventions aimed at preventing these errors are likely to result in cost reduction. Possibilities for the reduction of prescription errors are the use of electronic prescribing systems and clinical pharmacy services^(18,19)^. Our institution has an electronic prescription system and the beginning of a pharmacy residence program enabled the implementation of clinical activities for inpatients and outpatients with significant improvements in the hospital pharmacy unit.

One important breakthrough conquered in our institution during the study period was that the participation of pharmacists in daily clinical activities in inpatient units, which was essential to complement the activities of clinical pharmacists. This insertion allows the identification of DTPs that were not yet perceived in the pharmacy unit, such as the presence of interactions and incompatibilities between the solutions administered by Y catheter; inadequate protection or medication storage and infusions; problems in the interpretation of medications in the hospital's computerized information system.

The importance of the clinical pharmacist in the prevention, early detection and resolution of DTPs has become clear. The large number (n=933) of interventions performed can confirm the benefit of the pharmacist's involvement in the clinical activities. Approximately one in every seven prescriptions had some type of DTP, requiring a pharmacist intervention. This result is similar to that found by Franklin et al.^(8)^, which showed an error rate of 14.7% in their study. In our study, the clinical pharmacist has shown particularly the importance of individualized pharmacotherapy, which can be inferred from the most prevalent DTPs and CPIs, namely, dose and individualized/correct dosing. Other studies also detected the need of dose adjustment as the most frequent medication error^(20,21)^.

The presence of inappropriate/unnecessary medication and its related pharmaceutical intervention to suspend the medication also showed high prevalence. LaPointe^(22)^, in his review, showed as the most frequent medication errors: wrong medication (36.0%) and wrong dose (35.3%), similar to the results found in HC-UFPR.

Also, in relation to the most frequent DTPs, some situations need to be mentioned: the majority of the study population was comprised of critically ill patients (Adult ICU and Cardiologic ICU) and in this group the incidence of acute renal failure is high, reaching 23%, thereby justifying the need for dose adjustment of medications^(23)^. Moreover, the absolute prevalence of polypharmacy and the number of medications per prescription was high (average of 11 medications per order) also predisposing to a higher prevalence of inappropriate or unnecessary medications.

The medications predominantly involved in DPT were ranitidine, enoxaparin and meropenem. These medications are commonly prescribed to critically ill patients, for being part of clinical protocols (for example: ranitidine for prophylaxis of stress ulcer, enoxaparin for prophylaxis of deep venous thrombosis and enoxaparin for treatment of acute coronary syndrome) or for being used to treat pathologies frequent in this population (for example: meropenem for infections by *Gram*-negative bacteria).

The acceptability of the interventions made in the period was 76.32% (74.71% accepted and 1.61% accepted with alterations). It is important to consider that, in our study, pharmacist recommendations to physicians regarding pharmacotherapy monitoring, which correspond to 6.32% of the total, were registered only as educational actions, therefore without a measure of acceptability. This aspect may have led to a reduction in the acceptability rate of the study. A similar study conducted by Néri in a large university hospital in Ceará, Brazil, demonstrated an acceptability of 88.66% of the pharmacist interventions performed within 1 month^(24)^. In other hand, in a study performed by Leape et al., acceptance was 99%, while in another study published by Charpiat et al., acceptability rate was only 47%^(15,25)^.

During the classification of DTPs, several questions emerged, and they were discussed in weekly meetings between the team of clinical pharmacists and preceptorship. Through these discussions, it was possible to identify needs for adjustments in several steps, including: review of the standardization of pharmacist interventions and monitoring registration methods; periodic review of the clinical pharmacy manual; training and capacity building of first-year pharmacist residents, pharmacy technicians, members of the nursing staff, and medical staff; in addition to updating the dispensing routine.

Regarding the disclosure of the data collected, continuous reports of the clinical pharmacy performance were sent to the responsible units, assistance direction, teaching direction, and clinical direction of the hospital. This structure provided a wide dissemination of the activities performed, and permitted the assistance teams to discuss results. It allowed the identification of the most prevalent interventions, and the definition of potential improvement actions with the unit's responsible and the residence team to reduce these numbers.

Our study has some limitations. The prevalence of DTPs and pharmacist interventions collected could do not reflect the whole reality of our hospital, considering that the three different clinical units evaluated account for less than 10% of the total number of units. Otherwise, we could evaluate more than 6,000 prescriptions in the area of cardiovascular and critical care. In our experience, these units correspond to the most important areas regarding the occurrence of medication errors. Another limitation corresponds to the fact that the assessment of prescriptions was performed in the hospital pharmacy unit, often hampering the communication with the healthcare team and the perception of errors associated with the preparation and medication administration routine. Despite the fact that pharmacist participation in clinical rounds could minimize this limitation, we cannot rule out the possibility that DTP prevalence may have been underestimated.

Like any new process, the effective action of the clinical pharmacist in Brazil still has a long way to go. However, everyday the need for the inclusion of clinical pharmacists in healthcare teams becomes more evident, since the incidence of medication errors is still alarming, and pharmacist interventions can generate direct benefits for patient safety, as well as provide improvement in the quality of care. Furthermore, the process of medication use is a dynamic process and the interventions made by the clinical pharmacist can bring enhanced outcomes, thus ensuring better safety, efficacy and cost-effectiveness of pharmacotherapy.

## CONCLUSION

We evidenced that the review of prescription orders plays a key role in the activities of hospital clinical pharmacists and can contribute to improve the quality of medication use and patient safety. The data confirms that up to 14.6% of the prescriptions reviewed had some DTP, and interventions by pharmacists promoted beneficial changes in seven to ten prescriptions with some clinical problem. Moreover, these activities demonstrated that they improve communication inside the healthcare team and between pharmacists and patients.
